# Prognostic relevance of contrast extravasation in patients undergoing endovascular embolization of acute bleeding

**DOI:** 10.1007/s00330-025-11986-3

**Published:** 2025-09-06

**Authors:** Hans-Jonas Meyer, Simon Riegelbauer, Matthias Mehdorn, Hans-Michael Tautenhahn, Uwe Scheuermann, Silke Zimmermann, Sebastian Ebel, Timm Denecke, Manuel Florian Struck

**Affiliations:** 1 Department of Diagnostic and Interventional Radiology, University of Leipzig, Leipzig, Liebigstr. 20, 04103 Leipzig, Germany; 2 Department of Visceral, Transplantation, Thoracic, and Vascular Surgery, University Hospital Leipzig, Liebigstr. 20, 04103 Leipzig, Germany; 3 Institute of Laboratory Medicine, Clinical Chemistry, and Molecular Diagnostics, University Hospital Leipzig, 04103 Leipzig, Germany; 4 Department of Anesthesiology and Intensive Care Medicine, University Hospital Leipzig, Liebigstr. 20, 04103 Leipzig, Germany

**Keywords:** Acute bleeding, CT, Embolization, Massive transfusion

## Abstract

**Objectives:**

Contrast extravasation on imaging studies is a clinical surrogate for bleeding severity. However, the prognostic relevance of this imaging sign needs to be evaluated. The aim of this study was to analyze the impact of contrast extravasation defined by computed tomography (CT) and angiography on massive transfusion and 30-day mortality in patients with acute bleeding undergoing transarterial embolization (TAE).

**Materials and methods:**

A mixed cohort of patients with acute bleeding requiring treatment with TAE between 2018 and 2022 was retrospectively evaluated. All patients underwent triphasic CT to localize the source of bleeding and to calculate extravasation volumes in the arterial and portal venous phases. The bleeding rate *k* was calculated from the CT images.

**Results:**

A total of 128 patients (79 male, 61.7%) with a mean age of 67.4 years (range 21*–*95 years) and an all-cause 30-day mortality rate of 34.4% were included in the present analysis. A moderate positive correlation was identified between transfused packed red blood cell units and bleeding rate *k* (*r* = 0.33, *p* < 0.001). However, no correlation was found between transfused packed red blood cell units and arterial and portal venous extravasation volume. In multivariable logistic regression analysis, bleeding rate *k* was identified as an independent prognostic factor for massive transfusion (OR 25.77, 95% CI 1.35–493.61, *p* = 0.031, area under the receiver operating characteristic curve (AUROC) of the model: 0.847) and 30-day mortality (OR 25.04, 95% CI 2.29–273.42, *p* = 0.008, AUROC of the model: 0.781).

**Conclusion:**

CT-defined bleeding rate, k, is a prognostic factor for massive transfusion and 30-day mortality in patients with acute bleeding undergoing TAE and may be superior to the volume of contrast extravasation volume alone. Further studies are needed to confirm this finding.

**Key Points:**

***Question*** *Does contrast media extravasation on CT have a prognostic role in patients with acute bleeding?*

***Findings*** *Bleeding rate, k, was identified as an independent prognostic factor for massive transfusion (OR 25.77) and 30-day mortality (OR 25.04)*.

***Clinical relevance*** *Diagnostic triphasic CT can be used to provide prognostic information of patients with acute bleeding*.

**Graphical Abstract:**

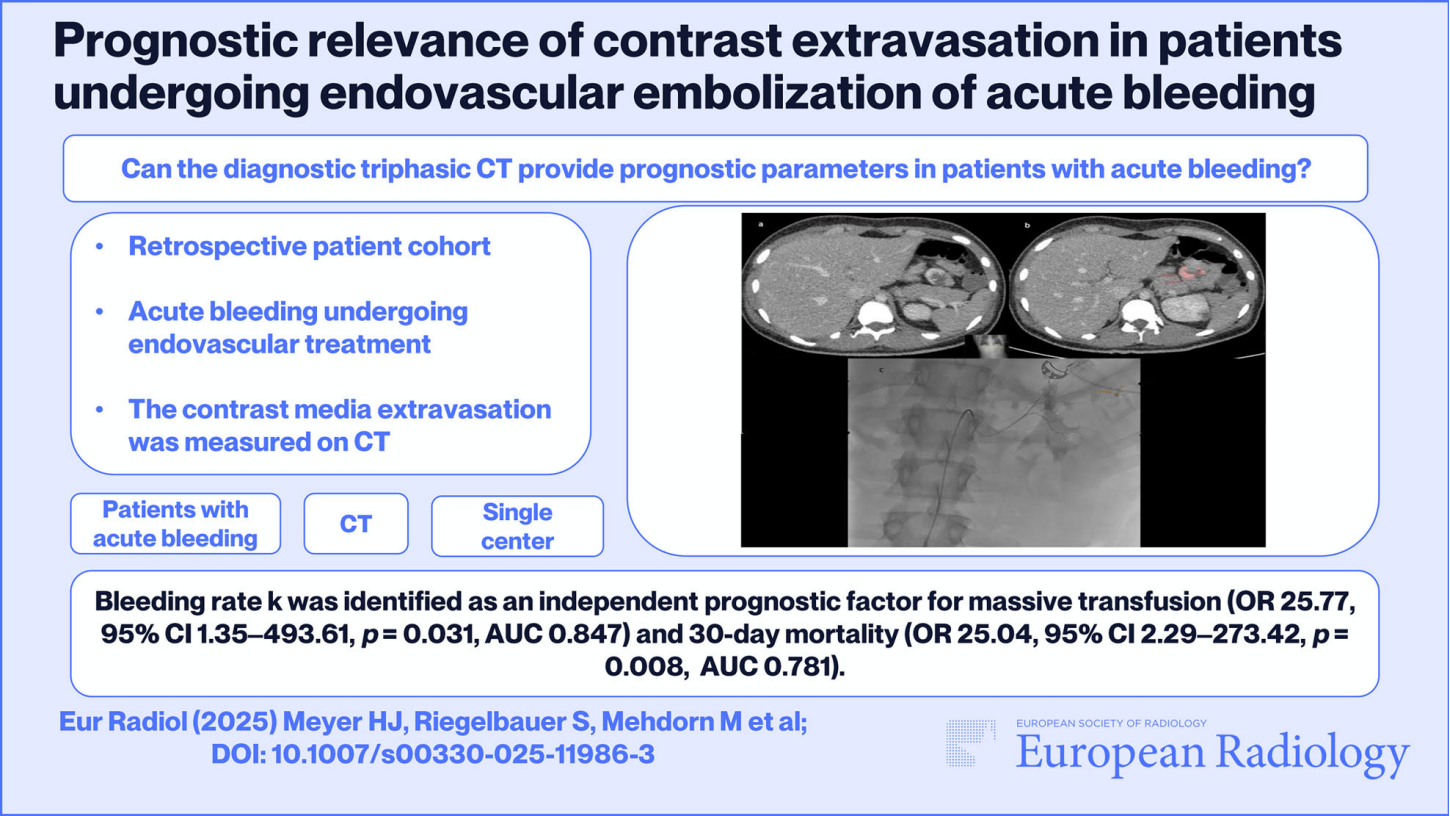

## Introduction

Acute bleeding is a life-threatening condition with a reported high mortality rate [1–3].

Immediate endovascular embolization of the bleeding source is a highly effective and less invasive treatment modality compared to emergency surgery [3–8]. A technical effectiveness rate of more than 95% was reported, which highlights the high clinical success rate. Endovascular treatment can be performed at almost any part of the human body in specialized centers. However, it must be discussed that despite the high technical efficacy, the clinical outcome of patients can be highly variable. Therefore, there is a need for effective predictive imaging markers to better stratify patients.

Nowadays, almost every patient with acute bleeding undergoes multi-slice computed tomography (CT) scan to localize the bleeding site and plan the endovascular procedure [9–11]. CT has been reported to have a sensitivity of almost 95% for detecting clinically relevant acute bleeding [9–11]. However, there are limitations such as very low bleeding rate or venous bleeding sites that have lower sensitivity on CT [12, 13].

There is a growing trend to use CT images not only for diagnostic purposes, but also to define image-based prognostic parameters and scores to better assess patients and predict disease outcome [14, 15]. Since almost every patient with acute bleeding undergoes CT, it could be crucial to extract prognostic factors from the images as a by-product.

A recent study investigated the prognostic significance of contrast extravasation defined by CT images with promising results in patients with upper and lower gastrointestinal bleeding [16]. The authors used the arterial phase and performed volumetry of contrast extravasation. It has been hypothesized that the amount of contrast extravasated from the vessel in a given time is a strong surrogate marker of bleeding severity, which also correlates with hemostatic therapy and massive transfusion. However, no other study has validated the formula used by the authors, and the significance of contrast extravasation volume in patients with bleeding from other localizations remains unclear. A more thorough examination and validation of these aspects is necessary to substantiate the prognostic significance of contrast extravasation in patients with acute bleeding.

Therefore, the aim of the present study was to investigate the prognostic relevance of CT- and angiography-defined contrast extravasation in patients with acute bleeding undergoing transarterial embolization (TAE).

## Materials and methods

### Patient acquisition

Prospectively collected data from all patients who underwent TAE for acute hemorrhage at our tertiary referral center between 2018 and 2022 were retrospectively analyzed for this project. The study was performed after approval by the local ethics committee (No. 25/21 and 319/24-ek) in accordance with the ethical standards of the institutional and/or national research committee and with the Helsinki Declaration of 1964 and its subsequent amendments or comparable ethical standards.

Inclusion criteria were available CT images obtained within 24 h prior to angiography and the TAE procedure performed. Patients were excluded if imaging or clinical data were not within the reported time frame, and CT imaging was performed without a contrast agent. The definition of acute bleeding encompasses cases that exhibit the following criteria: (I) Clinical symptoms of bleeding with a decrease in hemoglobin (Hb) concentration of ≥ 16.1 g/L (1 mmol/L) within 24 h. (II) The manifestation of symptoms consistent with hemorrhagic shock. (III) The presence of uncontrolled bleeding as evidenced by endoscopy.

The primary endpoint of this study was defined as 30-day all-cause mortality. The mortality rate was evaluated in days following the diagnosis of active bleeding, as determined by CT imaging. The secondary endpoint was the necessity for massive transfusion due to bleeding from the identified vascular lesion, which was localized by CT scan 24 h before and during angiography.

### Clinical parameters

Analysis of comorbidities in patient’s history included sex, age, body mass index (BMI), pre-injury medication of antiplatelet agents, heparin, and anticoagulants (warfarin or direct oral anticoagulants), Intervention during night (8 p.m.–7 a.m.), presence of shock (defined as rapid clinical deterioration with signs of impaired microcirculation (acidosis), ongoing need for continuous administration of vasopressors (norepinephrine) and transfusion of blood products), number of transfused packed red blood cell (PRBC) units, massive transfusion (defined as ≥ 10 units of PRBC within 24 h or ≥ 5 units of PRBC within 4 h [17, 18], systolic blood pressure, heart rate, Hb (mmol/L), platelet count (x10^9^/L), activated partial thromboplastin time (aPTT) (s), and prothrombin time (%). Localization of bleeding was stratified by body region.

### Transarterial embolization

TAE was performed in patients with acute arterial bleeding refractory to conservative, surgical, or endoscopic management and cases of recurrent bleeding. The decision to embolize was made by a multidisciplinary team based on clinical symptoms, evidence of blood extravasation on endoscopy, contrast-enhanced CT or digital subtraction angiography (DSA), and clinical chemistry. TAE was performed by board-certified interventional radiologists with at least 5 years of experience in interventional radiology. Access was usually via a transfemoral approach and rarely via a transbrachial approach. An initial angiogram was obtained with a 4 F or 5 F macrocatheter, depending on the target vessel. Target vessels were catheterized with 2.4 F or 2.7 F Progreat microcatheters (Terumo Medical). In most cases, the bleeding site was identified by direct detection of contrast extravasation on DSA. In some patients, empirical embolization was performed, taking into account indirect bleeding signs (e.g., tumor blush, vessel discontinuity, or vasospasm), CTA findings, and the location of vascular clips placed during endoscopy. The embolic agent was selected based on vascular anatomy, local hemodynamics, microcatheter stability, mechanism of bleeding, and the experience of the interventionalist. Embolic materials and devices used for TAE included embolization particles (Contour PVA; Boston Scientific), microspheres (Embozene; Boston Scientific), sponge (Gelaspon; Bausch+ Lomb), n-butyl-2-cyanoacrylate adhesive (Histoacryl; B. Braun), liquid embolic systems (Onyx; Covidien), 0.018” microcoils (Hilall or Tornado (SEF and LEF); Cook Medical), vessel plugs (Amplatzer; St. Jude Medical). These different materials and devices were used either alone or in combination, depending on the clinical situation and the preference of the interventionalist.

### Imaging technique

Contrast-enhanced, triphasic (native, arterial and portal-venous) CT was performed in a clinical setting using a 128-slice CT scanner (Ingenuity 128, Philips). In each case, the indication for the CT was to locate the source of bleeding. Intravenous iodinated contrast agent (90 mL Imeron 400 MCT, Bracco Imaging Germany GmbH) was administered at a rate of 4.0 mL/s via a peripheral venous line. Automatic bolus tracking was performed in the descending aorta with a trigger of 100 Hounsfield units (HU). Typical imaging parameters were: 100 kVp; 125 mAs; slice thickness, 1 mm; pitch, 0.9.

### CT contrast extravasation

In each case, contrast extravasation was calculated using the volume tool of the Philips advanced visualization workspace (version 15, Philips Healthcare). A trained radiology resident with 3 years of experience performed all measurements blinded to the clinical outcome. Acute bleeding was defined by the presence of contrast in the arterial phase, new in the native phase, and either dilated or morphologically altered in the portal-venous phase [19]. Extravasation volume was measured by selecting a region of interest within the contrast depot. Pixels above a threshold attenuation of 100 HU and below a threshold of 700 HU were labeled as extravasation, and the total volume (mL) and mean attenuation (HU) of the segmented extravasation volume were calculated. A lower limit of 100 HU, which also corresponds to the lower limit of extravasation attenuation [17], was chosen to exclude hematoma and other intrinsically hyperattenuating bowel contents. An upper limit of 700 HU was chosen to exclude bony or metallic structures. In addition, attenuation of the abdominal aorta at the level of the superior mesenteric artery was measured in the arterial phase axial plane to approximate blood pool attenuation. This was done separately in the arterial and portal-venous phases. The measurements were performed as described by Tse et al [16]. The bleeding rate *k* was then calculated according to the proposed formula by Tse et al The formula can be expressed as *k* = *V*_*E*_*c(t)/tc*_*i*_*(t); t* time, *v*_*E*_ (total extravasation volume; mL) *c*_*i*_(*t*) extravasation iodine concentration. Briefly, *k* is a function of extravasation volume, extravasation attenuation, aortic attenuation, and elapsed time [16]. The proposed cut-off value of > 0.8 mL for the arterial phase was evaluated as a prognostic factor [16].

For interreader agreement a senior radiologist with 9 years of general experience measured randomly selected subset of 30 patients again blinded to the results of the first measurement.

**Fig. 1:**
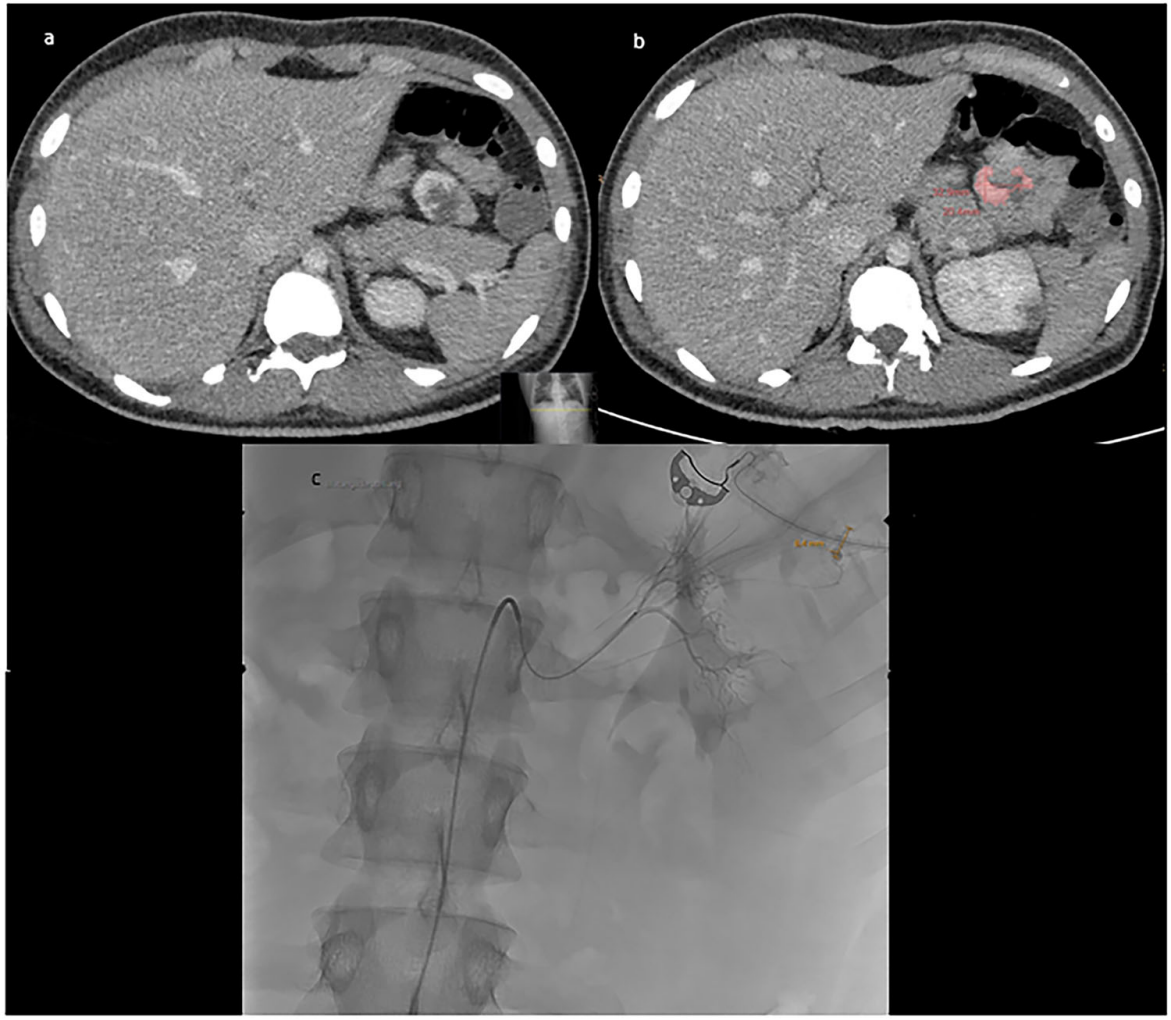
Representative male patient of the present cohort with an upper gastrointestinal bleeding. **a** Axial portal-venous phase image with the contrast media extravasation in a jejunal loop. **b** The measurement of the contrast media extravasation is highlighted in red. **c** The following endovascular procedure before embolization of the vessel

Figure 1 shows a representative case of the patient cohort to demonstrate the measurement of contrast extravasation.

### Statistical analysis

The collected data were analyzed using descriptive statistics (absolute and relative frequencies). The Shapiro–Wilk test was used to test the normality of the distribution. Spearman’s correlation coefficient (*r*) was used to analyze associations between the parameters studied. Group differences were calculated using the Mann–Whitney *U-*test and Fisher’s exact test, when appropriate. Univariate and multivariable logistic regression analyses were used to test for the effect of contrast media parameters on 30-day mortality and massive transfusion. For multivariable analysis, the rule of at least ten events per variable was applied to reduce the risk of confounding due to small sample size. The area under the receiver operating characteristic curve (AUROC) was reported in multivariable models. Interreader agreement was calculated using the intraclass coefficient (ICC) analysis.

In all cases, *p*-values < 0.05 were used to demonstrate statistical significance. Statistical analysis and graph generation were performed with GraphPad Prism 10 (GraphPad Software), SPSS (IBM, Version 25.0) and DATAtab (DATAtab e.U.).

## Results

During the observation period, a total of 258 patients underwent angiographic bleeding control, of whom 158 had arterial phase data available. Of these, 30 patients were excluded due to incomplete or missing data.

A total of 128 patients (79 male, 61.7%) with a median age of 67.4 years (range 21–95 years) and a 30-day mortality rate of 34.4% (44 patients) were included in the analysis. Table 1 summarizes the demographics of the patient population.

**Table 1:** Demographic characteristics of the patient cohort related to 30-day mortality

Parameters	All patients (*n* = 128)	Survivors (*n* = 84)	Non-survivors (*n* = 44)	*p*-value
Clinical parameters				
Sex (male)	79	43 (38.4%)	20 (40.7%)	0.87
Age (years)	67.4 (55.7–77.4)	70.5 (60.6–78.1)	66.5 (54.5–75.4)	0.168
BMI	25.8 (22.5–28.7)	24.9 (22.5–28.8)	25.9 (22.9–28.2)	0.857
Intervention during night (8 p.m.–7 a.m.)	40 (31.3%)	27 (32.1%)	13 (29.5%)	0.842
Shock	69 (53.9%)	36 (42.9%)	33 (75%)	**0.001**
Heart rate (1/min)	87 (75–101)	85 (74.8–100)	90 (75–105)	0.181
Systolic blood pressure (mmHg)	119 (98–141)	120 (96.8–142.3)	115 (100–132.2)	0.512
Transfusion of PRBC	89 (69.5%)	53 (63.1%)	36 (81.8%)	**< 0.001**
Number of transfused PRBC	3 (0–9)	2 (0–5.3)	6.5 (2–12.3)	**0.002**
Massive transfusion	42 (32.8%)	20 (23.8%)	22 (50%)	**< 0.001**
Laboratory parameters				
Hemoglobin (mmol/L)	4.8 (4.1–5.9)	5.1 (4.2–6)	4.4 (3.8–5.7)	**0.028**
Platelet count (x10^9^/L)	188 (118–289)	185.5 (118.8–309)	189 (123–275)	0.996
aPTT (s)	29.7 (26.2–37.9)	27.9 (25.2–32.6)	33.8 (30.5–55.8)	**< 0.001**
Prothrombin time (%)	71 (58–86.3)	75 (65–90)	64 (49.3–73)	**< 0.001**
Imaging parameters				
Arterial extravasation volume (mL)	1.38 (0.54–2.95)	1.15 (0.53–3.1)	1.56 (0.59–2.61)	0.486
Portal-venous extravasation volume (mL)	3.23 (1.33–7.64)	2.51 (1.01–6.5)	4.76 (1.79–12.46)	**0.021**
Bleeding rate *k* (mL/min)	0.06 (0.02–0.14)	0.05 (0.02–0.1)	0.07 (0.03–0.27)	**0.037**

*BMI* body mass index, *aPTT* activated partial thromboplastin time, *PRBC* packed red blood cell units; all values are the median (interquartile range) and number (percentage). Bold numbers indicate statistical significance (*p* < 0.05)

The majority of patients (109 patients; 85.2%) were treated with an antiplatelet and/or anticoagulant therapy (83 patients: heparin or low-molecular-weight heparins, 37 patients: aspirin and derivatives, 12 patients: direct oral anticoagulants, one patient: warfarin) and received continuous infusion of vasopressors (79 patients; 61.7%), with 69 patients (53.9%) being categorized as being in circulatory shock. Eighty-nine patients (69.5%) received PRBC transfusion, of which half (44 patients) received a mass transfusion.

The anatomic location of the bleeding was: thoracolumbar or abdominal wall in 26 cases (20.3%), lower gastrointestinal tract (GI) in 20 cases (15.6%), upper GI in 20 cases (15.6%), liver in 20 cases (15.6%), pelvis in 15 cases (11.7%), kidney in 12 cases (9.4%), spleen in 8 cases (6.3%), lower extremity in 4 cases (3.1%), pancreas in 2 cases (1.6%), and pulmonary artery in one case (0.8%). Table 2 presents all anatomic locations related to massive transfusion and 30-day mortality.

**Table 2:** Anatomical bleeding source, massive transfusion, and 30-day mortality

Bleeding source	Total number	Massive transfusion (yes)	(no)	30-day mortality (yes)	(no)
Thoracolumbar or abdominal wall	26	10	16	14	12
Lower GI	20	7	13	4	16
Upper GI	20	6	14	7	13
Liver	20	7	13	11	9
Pelvis	15	5	10	3	12
Kidney	12	3	9	2	10
Spleen	8	1	7	2	9
Lower extremity	4	2	2	0	4
Pancreas	2	1	1	0	2
Pulmonary artery	1	0	1	1	0

*GI* gastrointestinal tract

TAE was considered technically successful in every case defined by the angiographic results at the end of the procedure.

### Interreader agreement

The interreader agreement was substantial for all investigated imaging parameters for the volume of the contrast media extravasation and for the bleeding rate *k* with ICC values of 0.97 (95% CI 0.95;0.98) and 0.98 (95% CI 0.96;0.99), respectively.

### Correlation analysis between contrast extravasation and clinical parameters

There was a moderate positive correlation between bleeding rate *k* and the number of PRBC units transfused (*r *= 0.31, *p* < 0.001), whereas arterial and portal-venous extravasation volumes showed no correlation (*r* = 0.06, *p* = 0.507 and *r* = 0.17, *p* = 0.76, respectively). There was a weak inverse correlation between bleeding rate *k* and platelet count (*r* = −0.19, *p* = 0.031). No correlation was found between contrast agent parameters and age, BMI, systolic blood pressure, heart rate, Hb level, aPTT level and prothrombin time level.

### Associations with massive transfusion

The median bleeding rate *k* was significantly higher in 42 patients who received massive transfusion compared to 86 patients who did not (0.05 mL/s vs. 0.08 mL/s, *p* = 0.001), while the median arterial and portal-venous extravasation volumes were comparable in both groups (1.39 mL vs. 1.22 mL, *p* = 0.467, and 2.51 mL vs. 4.91 mL, *p *= 0.067, respectively).

In univariable logistic regression analysis yielded a statistically significant association between bleeding rate *k* and massive transfusion (OR 55.82, 95% CI 3.91–796.57, *p* = 0.003), which remained significant after adjustment for three other univariable significant variables: presence of shock, Hb level, and aPTT level (OR 25.77, 95% CI 1.35–493.61, *p* = 0.031, AUROC of the model: 0.847) (Table 3a).

**Table 3:** Logistic regression analyses of associations with massive transfusion and 30-day mortality

Parameter	Univariable OR	95% CI	*p*-value	Multivariable OR	95% CI	*p*-value
**(a) Massive transfusion** (***n*** = **42 patients)**						
Age	0.98	0.96–1.01	0.192			
Shock	16.85	5.5–51.68	**< 0.001**	11.58	3.65–36.71	**< 0.001**
SBP	0.99	0.98–1.01	0.304			
Hb	0.59	0.42–0.83	**0.002**	0.7	0.46–1.07	0.096
PLT	1	1–1	0.18			
aPTT	1.02	1–1.03	**0.034**	1.01	0.99–1.03	0.502
PT	0.98	0.96–1	0.055			
Arterial volume	1.02	0.98–1.05	0.337			
Portal-venous volume	1.02	0.99–1.06	0.226			
Bleeding rate *k*	55.82	3.91–796.57	**0.003**	25.77	1.35–493.61	**0.031**
**(b) 30-day mortality** (***n*** = **44 patients)**						
Age	1.02	0.99–1.05	0.139			
Shock	4	1.78 - 8.97	**0.001**	2.4	0.97–5.92	0.058
Systolic blood pressure	1	0.99–1.02	0.673			
Hemoglobin	0.72	0.53–0.98	**0.036**			
Platelet count	1	1–1	0.837			
aPTT	1.04	1.01–1.07	**0.002**	1.03	1.01–1.06	**0.018**
Prothrombin time	0.96	0.94–0.98	**< 0.001**	0.98	0.95–0.99	**0.033**
Arterial volume	0.99	0.96–1.03	0.655			
Portal-venous volume	1	0.98–1.03	0.651			
Bleeding rate *k*	21.24	2.02–223.57	**0.011**	16.31	1.43–185.84	**0.025**

*HR* hazard ratio, *CI* confidence interval, *shock* defined as progressive vasopressor administration, blood transfusion requirement, and acidosis, *aPTT* activated partial thromboplastin time, *arterial volume* contrast extravasation volume in the arterial phase, *portal-venous volume* contrast extravasation volume in the portal-venous phase.

In multivariable analysis, the rule of ten events per variable was applied, resulting in a maximum of four possible predictors of massive transfusion and 30-day mortality. The most significant variables were included in the multivariable analysis. Bold numbers indicate statistical significance (*p* < 0.05). Model area under the receiver operating characteristic curve (AUROC) for massive transfusion (a) 0.847, and for 30-day mortality (b) 0.797

Arterial and portal-venous extravasation volumes were not associated with massive transfusion in univariable logistic regression analysis (OR 1.02, 95% CI 0.98–1.05, *p* = 0.337 and OR 1.02, 95% CI 0.99–1.06, *p* = 0.226, respectively). Using the arterial volume cut-off of > 0.8 mL, there was also no association with massive transfusion (OR 1.24, 95% CI 0.58–2.65, *p* = 0.588).

### Associations with all-cause 30-day mortality

The median portal-venous extravasation volume and the median bleeding rate *k* were significantly higher in 44 non-survivors compared to 84 survivors (2.54 mL vs. 4.76 mL, *p* = 0.021, and 0.05 mL/s vs. 0.07 mL/s, *p* = 0.037, respectively). Arterial extravasation volume was similar in both groups (1.15 mL vs. 1.56 mL, *p* = 0.486).

In univariable logistic regression analysis, bleeding rate *k* was statistically significantly associated with 30-day mortality (OR 21.24, 95% CI 2.02–223.57, *p* = 0.011), which remained significant after adjustment for three other univariable significant variables: presence of shock, aPTT level, and prothrombin time level (OR 16.31, 95% CI 1.43–185.84, *p* = 0.025, AUROC of the model: 0.797) (Table 3b).

Arterial and portal-venous extravasation volumes were not associated with 30-day mortality in univariable logistic regression analysis (OR 0.99, 95% CI 0.96–1.03, *p* = 0.655 and OR 1, 95% CI 0.98–1.03, *p* = 0.651, respectively). Using the arterial volume cut-off of > 0.8 mL, there was also no association with 30-day mortality (OR 1.61, 95% CI 0.75–3.46, *p* = 0.226).

Univariable logistic regression analysis of subgroups GI bleeding vs. non-GI bleeding and organ-bleeding vs. musculoskeletal bleeding revealed no significant differences regarding massive transfusion (OR 0.97, 95% CI 0.43–2.15, *p* = 0.934 and OR 0.83, 95% CI 0.38–1.78, *p* = 0.627, respectively) and 30-day mortality (OR 1.5, 95% CI 0.66–3.4, *p* = 0.332 and OR 0.79, 95% CI 0.37–1.69, *p* = 0.551, respectively).

## Discussion

The findings of the present study indicate that the bleeding rate, denoted by *k*, rather than the volumes of arterial and portal-venous extravasation, is associated with the necessity for mass transfusions and 30-day mortality.

There is a growing trend to use images obtained for diagnostic purposes to extract quantitative prognostic parameters. Almost every patient with acute bleeding undergoes a pre-interventional triphasic technique to localize the exact bleeding site. Therefore, the rationale of the present analysis was that the amount of contrast extravasation defined by CT might be a predictor of bleeding severity and, therefore, prognostic in these patients. Improved characterization of patients at risk for a fatal outcome on initial CT may be critical to clinical care. Hypothetically, this could lead to a higher index of suspicion in patient management and more intensive treatment at an early stage or to a less restrictive use of blood and coagulation products in this special patient group.

The previous study by Tse et al evaluated 52 patients with life-threatening gastrointestinal bleeding, including 6 cases of upper gastrointestinal bleeding, 18 cases of small bowel bleeding, and 26 cases of lower gastrointestinal bleeding [16]. The main finding of this initial study was that higher extravasation volumes correlated with hemostatic therapy (*p* = 0.007), intraprocedural active bleeding (*p* = 0.003), and mass transfusion (*p* = 0.0001). However, there was no association with mortality (*p* = 0.936). The authors further proposed a threshold volume of 0.80 mL or greater to identify patients at risk, which resulted in an odds ratio for hemostatic therapy of 8.1 (95% CI, 2.1–26), active bleeding of 11.8 (2.6–45), and massive transfusion of 18 (2.3–65) [16]. Since then, the proposed formula for bleeding rate *k* and the proposed threshold of > 0.80 mL has not been systematically evaluated, and external validation is still missing.

In addition to confirming the association of bleeding rate *k* with massive transfusion, the present results now also suggest a prognostic relevance of bleeding rate *k* for 30-day mortality. This is of particular interest because no association was found for these parameters in the study performed by Tse et al Thus, the present study contradicts the previous study, which could not demonstrate a prognostic significance of contrast extravasation. Several reasons can be discussed to better reflect these aspects.

A recent study investigated the prognostic relevance of contrast extravasation in a heterogeneous cohort of patients with CT-detected extravasation of various etiologies [20]. The diameter of contrast extravasation greater than 8 mm had a strong prognostic relevance with an odds ratio of 2.34 (95% CI 1.23–4.45, *p* = 0.009) in univariable analysis and an odds ratio of 3.96 (95% CI 1.04–15.07, *p* = 0.043) in multivariable analysis [18]. The reported in-hospital mortality of 25.7% in this study was lower than that of 34.4% in the present study. However, it must be acknowledged that the patient sample of this study is more heterogeneous with different treatment options compared to the current study, with only endovascular patients [20].

Another study showed that the volume of contrast extravasation can predict the positive angiographic result to identify the exact localization of bleeding [21]. It can be hypothesized that this may also have an impact on the efficacy of the treatment with better embolization results and ultimately a better overall outcome for the patients. However, the study by Hsu et al did not further investigate mortality and technical outcomes [21].

Regarding the contrast media extravasation demonstrated by angiography, Lee et al studied patients with or without upper gastrointestinal bleeding with or without visible extravasation [22]. They showed that patients with active extravasation received more pRBC units (5.3 vs. 2.8 units, *p* < 0.001) and fresh frozen plasma (4.8 vs. 1.7 units, *p* = 0.005) transfusions 24 h before angiography and were more likely to be hemodynamically unstable at the timepoint of the procedure (67% vs. 28%, *p* = 0.001) than patients without active extravasation [22].

Before clinical translation, the possible generalizability and stability of the measurements have to be proven. Different CT acquisition protocols could influence the bleeding rate *k* in different aspects. The HUs for the aorta standardization can be influenced by the CT scanner, vendor and reconstruction algorithm. The time of the acquisition of the arterial phase is included in the formula and should therefore be a negligible factor. The measurement of the volume of the arterial phase extravasation is prone to reader bias, but it showed an excellent interreader agreement in our analysis. However, it remains unclear whether different readers with various experience levels can achieve the same results.

A multicenter evaluation of the investigated parameters using different CT scanners, protocols and readers is needed to test the general stability of the imaging markers.

The present cohort of patients is characterized by significant heterogeneity, with a variety of bleeding locations that are associated with varying mortality rates. When solely gastrointestinal bleeding is considered, this diversity may result in divergent conclusions. For example, it is known that patients with upper gastrointestinal bleeding tend to have a worse outcome than patients with abdominal wall bleeding [7, 8, 23].

In subgroup analyses according to the bleeding site, no differences could be observed in our cohort. One should nevertheless acknowledge that the present analysis reflects daily clinical routine with the occurrence of patients with active bleeding in all kind of body localizations.

It is noteworthy that the present mortality is rather high, indicating a high case severity. In comparison, Powerski et al reported a 30-day mortality of 18.4% in their cohort of 327 patients undergoing endovascular treatment [24]. The same study demonstrated that bleeding intensity and extent of injury were the only prognostic factors influencing clinical outcome [24]. However, this study did not evaluate the potential prognostic relevance of contrast extravasation.

Despite the high clinical and technical success of TAE, there is still a paucity of data on relevant prognostic factors in an unselected cohort of patients with acute bleeding undergoing TAE. The present study may add to the existing literature that contrast extravasation may be considered as a prognostic imaging marker. This should be further evaluated in more homogeneous cohorts to better adjust for potential confounding factors.

The current analysis has several limitations. First, retrospective single-center studies are prone to potential inherent bias. There may be center-specific preferences regarding the indication for TAE and possible treatment alternatives, such as emergency surgery. Hemodynamic measurements and laboratory values were significantly influenced by vasopressors, blood transfusions and anaesthetics and should be interpreted with caution. The majority of patients experienced bleeding events related to antiplatelet and anticoagulant medications. Patients without exposure to these substances (and their underlying conditions and diseases) may have different risk factors for massive transfusion and mortality. In addition, contrast agent quantification was performed by a trained reader blinded to the clinical outcomes. However, the resulting investigator bias is likely to be low because it was performed in a semiquantitative manner by contouring the sharply defined border defined by the contrast agent. Another limitation of the study is that we could not stratify the cohort according to the cause of death. Not all patients may have died due to volume loss but other causes like comorbidities. Not enough clinical data were available and the current patient sample is too heterogeneous to better adjust for this. It remains unclear whether the contrast media extravasation may be a stronger prognostic factor in patients with volume loss and hemorrhagic shock as the primary cause of death.

## Conclusion

The CT-defined bleeding rate *k* is a vital prognostic indicator for mass transfusion and 30-day mortality in a diverse cohort of patients with acute bleeding undergoing TAE treatment. It surpasses the significance of the arterial and portal-venous extravasation volume alone as a prognostic parameter. Further studies are essential to validate these findings and explore potential modifications in patient management.

## Abbreviations

AUROC: Area under the receiver operating characteristic curve
CT: Computed tomography
DSA: Digital subtraction angiography
HU: Hounsfield unit
TAE: Transarterial embolization
